# The College's Barometer of Progress: The Anti-College Party Steadily Loses Ground

**Published:** 1917-03-03

**Authors:** 


### THE MATRONS' AND SISTERS' DEPARTMENT.

### THE COLLEGE'S BAROMETER OF PROGRESS.

### The Anti-College Party Steadily Loses Ground.

The times in which we live, though specially onerous to the administrator and the worker, are full of interest for those with eyes to see and mind to understand. The Hon. Arthur Stanley set himself more than a year ago to secure the co-operation by united action of the nurse-training schools and trained nurses generally throughout the country in support of the immediate establishment of a Royal College of Nursing. As the nursing world knows, the result has been practically the unanimous support of the College by the British nursing profession, for those who the "*dividers*" claim to be dissentients in the profession cannot represent more than one out of every twenty fully-trained certificated nurses, if so many, and amount in the whole possibly to considerably fewer than 5,000 nurses. Trained nurses are exerting themselves to build up the Register of the College quickly, and they are registering in increasing numbers, the average being not less than 1,000 per month. These are remarkable figures, and demonstrate that the College Register at the present rate of progress must speedily become the National Register of Trained British Nurses.

Such a result reflects great credit upon the business judgment of British nurses generally, and proves their capacity spontaneously to co-operate in defence of themselves and their profession. *The Nursing Mirror* of February 24, page 401, published "The Dream of Number One," being the experience of a trained nurse, in which, in the course of her dream, she came to an architecturally beautiful building inscribed *The Royal College of British Nurses*, which she entered, and found an organisation so far-reaching and perfected as to fulfil to the full the aspirations of the most ambitious and far-seeing members of the nursing profession. This flight of imagination is noteworthy as marking the confidence which the whole body of British nurses have in the Hon. Arthur Stanley as their Parliamentary leader and friend. Mr. Stanley is one of the ablest and most trusted members of the House of Commons, and possesses a wide experience of affairs. He of all men is specially fitted to create a College, and to plan, organise, and finance it upon lines which will make it fulfil the ideal, so far as British Nurses are concerned, of a National College of All Souls, for which an Officer Wounded on the Somme made an eloquent plea in the *Times* of February 19. Mr. Stanley has nearly everything ready to complete his work so far as the Royal British College of Nursing is concerned, and has behind him, as he explained at Leeds, the influence and support of two great organisations which, in co-operation, can speedily raise the necessary funds to build the College and to endow it adequately.

The small party of nurses, or rather the lady who leads them who has deliberately attempted to divide the nursing profession in Great Britain, are busily engaged in dressing up Weakness and Inexperience with the aid of Figments and Imagination, to personate Wisdom and Representative Government for British Nurses. They now present them as the real article to the nursing profession and the public, though they have had a succession of warnings that British Nurses are not fools. British Nurses have made it plain that they are not going to suffer nonsense of this kind to delay for one moment the establishment of the College of Nursing, its speedy fruition and commanding success. The party of "Divide" have had continuous rebuffs which have exposed the hollowness of their contentions. These rebuffs include (1) the repudiation of the Central Committee by the Great Body of Scottish Nurses and Training Schools. (2) The memorable overthrow of the party of "Divide," which reduced them to silence at the special general meeting of the Royal British Nurses' Association which they attended, when the amalgamation with the College was adopted without a dissentient vote. (3) The meeting of the Manchester Branch of the National Union of Trained Nurses on the 10th ultimo, dealt with in The Hospital of February 17, page 409, which demonstrates the hollowness of the claim of the Central Committee to be a representative body and so superior to the Council of the Royal British College of Nursing, seeing that the National Union of Trained Nurses is one of the so-called representative bodies on the Central Council, the policy of the Executive Committee of which was censured and overthrown by the members of the most powerful branch of this Union, that of Manchester, in a meeting of eighty-six nurses, eighty-one of whom supported with acclamation the condemnation of the policy of opposition to the College.

The Germans have a way of reporting reverses and defeats as victories, or tactical and deliberate movements making for victory. This habit seems to have won the admiration to imitation of the Leader of the Central Committee, for we find the *British Journal of Nursing*, of February 24, reports a resolution of the Executive Committee of the Society for the State Registration of Trained Nurses (another of the representative bodies which modestly conceals in oblivion the names of its executive and a list of those present at its meetings), censuring Miss Sparshott, matron of the Manchester Royal Infirmary, for "advancing claims and an autocracy over nurses" which Miss Sparshott certainly does not advocate nor uphold. It is so easy to draft a resolution containing high-sounding phrases like that referred to, which, as in this instance, crumble to dust when examined with the actual facts of the Manchester meeting and the policy of the Council of the College, which Miss Sparshott supported. Miss Sparshott's fault in the eyes of the suffragette party, who seem to control the National Union of Trained Nurses at present, is that she upheld the highest ideals of the nursing profession which the Union was originally founded to maintain, and obtained the support of an overwhelming majority of the members for the Royal College of British Nursing.

The Leader of the Central Committee appears to have become lost in the fogs of make-believe in which she persists in dwelling. Sympathy and commiseration will be extended to her by everybody who reads the article, "Education is a Spiritual Activity," in the *British Journal of Nursing* of 24th ultimo. After deliberately devoting all her strength to an effort to break up the British Nursing Profession so as to deliver it, tied and bound, to the tender mercies of the Central Committee, the Leader refers to the nurses present at the meeting on January 18, of whom she was one, when the R.B.N. Association united with the College, as "those rows of faces" which made the beholder "realise the absence of the spirit in the dumb body." She declares, and we agree, though for totally different reasons, "those nurses who love spiritual freedom and fight for it in the organisation of their profession may take heart of grace." They may indeed, for the Nurses who have and are registering with the Royal College of British Nursing are "organising in defence of their Charter of Professional Liberty," which they have obtained by the amalgamation of the College and the Royal British Nurses' Association. The Leader, however, did not, we presume, mean, as the *Journal's* article clearly enforces, that this organisation of the Royal College of British Nursing is a striking proof that "the material never yet overcame the spiritual." Whatever her meaning, "those nurses who love spiritual freedom and fight for it, in the organisation of their profession, may take heart of grace," for they can have no doubt of the fact, that the strength of the confidence of the British Nursing Profession in the Royal College of Nursing is striking proof that "the material never yet overcame the spiritual."

